# Chinese Herbal Medicine for Myasthenia Gravis: A Systematic Review and Meta-Analysis

**DOI:** 10.3389/fphar.2018.00969

**Published:** 2018-08-30

**Authors:** Shuang Chen, Meng-Bei Xu, Xiao-Li Zhou, Pei-Qing Rong, Ting-Yu Jin, Guo-Qing Zheng

**Affiliations:** Department of Neurology, The Second Affiliated Hospital and Yuying Children’s Hospital of Wenzhou Medical University, Wenzhou, China

**Keywords:** myasthenia gravis, Chinese herbal medicine, systematic review, meta-analysis, T regulatory cells

## Abstract

Myasthenia gravis (MG) is an acquired autoimmune disease with the disorder of the neuromuscular junction transmission caused by autoantibodies. Currently, various Chinese herbal medicines (CHMs) are widely used for MG. This meta-analysis was conducted to assess the effectiveness and safety of CHMs for MG and its possible mechanisms. Fourteen studies with 1039 individuals were identified by searching seven databases from inception to March 2017. The methodological quality was assessed by using 7-item criteria from the Cochrane’s Collaboration tool, and which assessed ≥4 “yes” in the domains were selected for detailed assessment and meta-analysis. All the data were analyzed using Rev-Man 5.3 software. Meta-analysis showed a significant effect of CHM as adjuvant therapy for improving the effectiveness compared with WCM alone or placebo in treating MG (*p* < 0.01). Moreover, there were fewer adverse effects and relapse rate in total when compared with the control group. The possible mechanisms of CHM for MG are associated with immunoregulation by reconstituting the functional ability of Tregs. In conclusion, despite the apparent positive results, the present evidence supports, to an extent, that CHM can be used for MG patients because of the methodological flaws and CHM heterogeneity. Further rigorous RCT for MG is needed.

## Introduction

Myasthenia gravis (MG) is an acquired autoimmune disorder of neuromuscular junction characterized by the easy fatigability and weakness of the skeletal muscles in which autoantibodies mostly directed to the acetylcholine receptor (AChR) and muscle-specific kinase (MuSK) (Gilhus and Verschuuren, 2015; Sanders et al., 2016) In addition, about 10–15% double-negative MG patients have antibodies against lipoprotein-related protein 4 (LRP4) (Li et al., 2018). The incidence rate of MG ranged from 0.3 to 3.0 per 100,000 worldwide (McGrogan et al., 2010). Epidemiological studies have shown an increasing incidence and prevalence of MG, especially among the elderly due to the diagnostic technique development (Zieda et al., 2018). Currently, the main treatments for MG include thymectomy, symptomatic and immunosuppressive (IS) treatments, and immunomodulating therapies such as intravenous immunoglobulin (IVIg) and plasma exchange (PLEX) (Sanders et al., 2016). However, there is no internationally accepted standard of care, and no one treatment best for all patients because of heterogeneous of MG (Sanders et al., 2016, 2018). In particular, orthodox therapy for effective symptom control often requires prolonged and even life-long IS treatment with debilitating adverse effects (Gotterer and Li, 2016). Furthermore, a proportion of refractory MG patients usually presented with the debilitating weakness, frequent relapses, MuSK or LRP4 antibody positive, post-thymectomy, and/or required high-dose steroids and add-on other IS agents (Drachman et al., 2008; Suh et al., 2013). Thus, the alternative strategy for MG or declining the side-effect of IS is urgently needed.

Traditional Chinese medicine (TCM), one of the holistic medical systems in the world, has a history of thousands years and now still used actively in China and elsewhere worldwide. In modern time, Chinese herbal medicine (CHM), one of main treatment modalities of TCM, is widely used for MG in clinic and obtained experimental evidence (Orhan, 2013; Cui et al., 2015). However, the current evidence available is insufficient to support CHM as a routine use for MG due to the poor methodological quality of the primary studies (Lyu and Sun, 2015). Thus, we conducted a systematic review of CHM for MG focused on the clinical evidence and possible mechanisms according to the high-quality randomized-controlled clinical trials (RCTs).

## Materials and Methods

Ethical approval was not required for literature research. The design, implementation, and reporting of outcomes of this study were conducted according to the Preferred Reporting Items for systematic reviews and meta-analyses: The PRISMA Statement (Moher et al., 2009) and our previous articles (Yang et al., 2017).

### Database and Search Strategies

Two reviewers independently searched the following databases: Chinese National Knowledge Infrastructure (CNKI), VIP Journals Database, Wanfang data Information Site, Chinese Biomedical Literature Database, PubMed, EMBASE, and Cochrane Library from inception to March 2017. The search strategy of PubMed was as follows, and was modified to suit other English or Chinese databases.

PubMed search strategy:

#1. myasthenia gravis [mh]
#2. Medicine, Chinese Traditional [mh]
#3. Herbal Medicine [mh]
#4. Integrative Medicine [mh]
#5. traditional Chinese medicine [tiab]
#6. herb^∗^ [tiab]
#7. or/2-6
#8. #1 and #7
#9. randomized controlled trial [pt]
#10. controlled clinical trial [pt]
#11. randomized [tiab]
#12. placebo [tiab]
#13. drug therapy [sh]
#14. randomly [tiab]
#15. groups [tiab]
#16. or/9–15
#17. animals [mh] not (humans [mh] and animals [mh])
#18. 16 not 17
#19. #8 and #18

### Eligibility Criteria

#### Types of Studies

Only high-quality RCTs that received at least four out of seven “yes” in the domains through Cochrane risk of bias (RoB) tool were selected, regardless of its blinding, language, or publication status. Quasi-RCTs in which allocation to treatment was obtained by alternation, the use of alternate medical records, date of birth, or other predictable methods were excluded.

#### Types of Participants

We included participants with a diagnosis of MG, according to Myasthenia Gravis Foundation of America (MGFA) recommendations for Myasthenia gravis clinical trials (2000) (Jaretzki et al., 2000), MGFA recommendations for Myasthenia gravis clinical trials (2012) (Benatar et al., 2012), International Consensus Guidance for the Management of Myasthenia Gravis (2015) (Sanders et al., 2016), 5th National Conference of Neuroimmunology (NCNI) for the diagnostic criteria of Myasthenia gravis (China, 1997) (Xu, 2000) and Chinese Medical Association of Neurology (CMAN) Expert Consensus for the diagnosis and treatment of Myasthenia gravis (2011) (Chinese Medical Association of Neurology Branch of neuroimmunology Group, 2011). The other diagnostic criteria with comparable definitions were also used. The NCNI criteria were as follows: (1) in clinic, fluctuating skeletal muscle weakness that progressively worsens during periods of physical activity and improves after periods of rest. Typically, the weakness and fatigue are worse toward the end of the day; (2) in pharmacology, acetylcholinesterase inhibitors (CHEIs) are an effective treatment for MG; (3) in clinical electrophysiology, the low-frequency repetitive nerve stimulation (RNS) decreased more than 10%, and single-fiber electromyography (SFEMC) reveals increased jitter; (4) in immunology, the positive AChR antibody contributes to diagnosis; (5) in pathology, if possible, it contributes to confirm the diagnosis, in which the post-synaptic muscle membrane is distorted and simplified, having lost its normal folded shape, and a decrease of concentration of AChRs on the muscle end-plate membrane (Xu, 2000). There were no restrictions on the gender, age, or race of patient. Patients belonged to allergy (allergic to more than two kinds of food or drugs), serious complications, such as cardiovascular diseases, renal insufficiency or other severe systemic diseases, and women in pregnant or lactating were not considered. Lambert–Eaton myasthenia syndrome was also excluded.

#### Types of Interventions

Analyzed treatments were CHM as monotherapy or adjuvant therapy in any dose or any forms. Comparator treatments were western conventional medication (WCM) or placebo. WCM refers to the combination of needed therapies of the following aspects (Skeie et al., 2010; Sanders et al., 2016): (1) general supportive care; (2) symptomatic therapies mainly with CHEIs such as pyridostigmine, edrophonium chloride, and neostigmine; (3) IS therapies such as glucocorticosteroids, azathioprine, Cyclosporin A (CYA), mycophenolate mofetil (MMF), Tacrolimus, methotrexate (MTX), cyclophosphamide, monoclonal antibodies, complement inhibition; (4) IVIg or PLEX; and (5) Thymectomy. Studies comparing one kind of CHM therapy with another CHM were excluded.

#### Types of Outcome Measures

The primary outcome measures included scales for assessing the extent of MG and muscle weakness using the Quantitative Myasthenia Gravis (QMG) scores (Barohn et al., 1998) and MG clinical absolute and relative scores (Wang et al., 1997). The MG clinical absolute and relative scores are a 60-point scale evaluate ptosis, eyelid fatigue, eye movement in the horizontal direction, right and left arm held outstretched at 90°, flexion of the knee and hip at 90°, facial muscles, chewing and swallowing, and respiratory muscle function (Wang et al., 1997). Each item is graded from 0 to 8 except facial muscles which score 0 to 4. Lower scores represent the better function.

The secondary outcomes included: (1) relapse rate after follow-up; (2) total clinical effective rate; and (3) adverse events. The total clinical effectiveness rate (Xu, 2003) was assessed at the end of the treatment using five grades as clinical cure (the related clinical symptoms were recovered up to 95–100%), markedly effective (the related clinical symptoms were recovered up to 80–95%), effective (the related clinical symptoms were recovered at 50–80%), improved (the related clinical symptoms were recovered at 25–50%), and invalid (the related clinical symptoms were recovered <25%).

### Selection and Data Extraction

Two reviewers independently identified the included studies and assessed the study eligibility. Reasons for the exclusion of studies were recorded. Information was extracted from the qualified articles by using a standardized data extraction form as follows: (1) general information: first author, the year of publication, and country; (2) characteristics of participants: sample size, age, gender, and disease duration; (3) methodological characteristics: study design, MG severity (Osserman classification), and diagnostic criteria; (4) details of intervention: type of intervention, duration of treatment, and follow-up time; and (5) outcome measures. Any disagreements were resolved by discussion with or by involving a third author.

### Assessment of Risk of Bias

The RoB of included studies was assessed by the Cochrane RoB tool with seven domains as follows: (1) generation of random sequence; (2) allocation concealment; (3) blinding of participants and personnel; (4) blinding of outcome assessment; (5) incomplete outcome data; (6) selective reporting; and (7) other bias, including sample size estimate, comparable baseline characteristic, and potential interests. The RCTs which received at least four “yes” in the domains were selected.

### CHM Composition

Specific herbs in the CHM formulae were recorded in **Table 2**. The frequency of use for a particular herb was calculated, and those used at a high frequency are described in detail.

### Description of Possible Mechanisms

Animal-based mechanism studies of active compounds from frequently used herbs in MG and related autoimmune disease were searched. The following information was recorded for such studies: first author’s name, publication year, the identity of active compounds and their herbal sources, experimental models used, intervention and control treatments, and suggested mechanisms.

### Statistical Analysis

Statistical analysis was performed by Cochrane Collaboration Review Manager Software (RevMan 5.3). Standard chi-square test and *I*^2^ statistic were used to examine the heterogeneity between trial and control results. A fixed effects model (*I*^2^ < 50%) or a random effects model (*I*^2^ > 50%) was used depending on the value of *I*^2^. The value of *p* < 0.05 was considered statistically significant. Dichotomous outcomes were calculated by the risk ratio (RR) with 95% confidence interval (CI), whereas continuous outcomes were summarized using standardized mean difference (SMD) with 95% CI. Publication bias was checked graphically by using the funnel plot, and approximately symmetric shows no existence of publication bias.

## Results

### Study Selection

**FIGURE 1:**
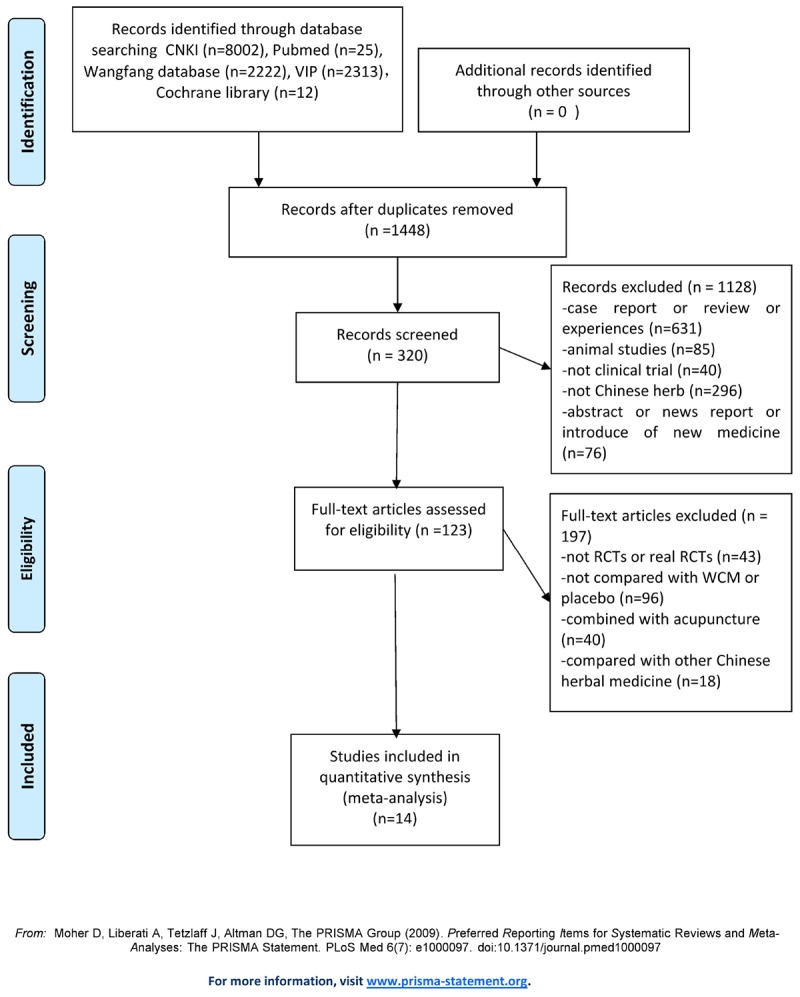
Flowchart of study screening.

A total of 2649 potentially relevant studies were identified in our search strategy from five databases, and 1201 duplicates were excluded. Of the rest 1448 articles, 1128 studies were removed by screening the titles and abstracts with the following reasons: (1) a case report or review, (2) not a clinical trial, (3) summary of clinical experiences, and (4) not Chinese herbal. By reading the remaining 320 full texts articles, we excluded 197 studies due to the following reasons: (1) not RCTs or not real RCTs, (2) combined with acupuncture, (3) not compared with WCM alone or placebo, and (4) retrospective study. Ultimately, 132 RCTs examining the efficacy of CHM for MG were included for qualitative analysis. Among them, 14 studies were assessed ≥4 domains with “yes” and selected for further assessment and meta-analysis. On the contrary, other 118 studies were excluded as being assessed ≥3 domains with “unclear” or “no.” The screening process is shown in a flow diagram (**Figure 1**).

### Study Characteristics

A total of 14 studies were finally included, published from 2003 to 2016. Only one study was published in English, and the rest of 13 studies were published in Chinese including eight master/doctorial theses. The sample sizes of the included studies ranged from 30 to 241, with a total of 1039 subjects, 521 patients in treatment groups, and 518 patients serving as controls. Among the 14 studies, no trial compared the CHM with placebo, and the comparisons of CHM alone with pyridostigmine bromide or corticosteroids were performed in three studies. CHM plus pyridostigmine bromide and corticosteroids vs. pyridostigmine bromide and corticosteroids were conducted in four studies, CHM plus pyridostigmine bromides. Pyridostigmine bromide in five studies, CHM plus corticosteroids vs. corticosteroids in two studies. The course of treatments lasted from 2 to 9 months. Adverse effects were reported in 10 studies, among which 2 studies reported no adverse events occurred. The characteristics of the 14 included studies were described in detail in **Table 1**. The constituent of CHM in each included study was listed in detail in **Table 2**.

**Table 1:** Characteristics of the included studies.

Included trials	Eligibility criteria	Study design	Intervention drugs		Sample size	Gender (male/female); Mean age (y)		Disease duration before treatment		Course of treatment	Outcome measure	Intergroup differences
									
			Trial	Control		Trial	Control	Trial	Control			
Niu, 2009	NCNI criteria	Multi-center RCT	HQFF+ PDN	Placebo + PDN	60	11/19; 43.20 ± 18.60	13/17; 43.20 ± 17.10	N.R.	N.R.	3m	1. Clinical absolute and relative scores 2. Clinical efficacy 3. Side effect	1. *p* < 0.05 2. *p* < 0.05
Jiang et al., 2014	NCNI criteria	Multi-center RCT	JJN + PB	Placebo+ PB	60	12/18; 48.70 ± 16.45	11/19; 43.20 ± 18.60	28.03 ± 23.83 (m)	30.43 ± 29.69 (m)	6m	1. QMG scores 2. Clinical efficacy (3m) 3. Side effect	1. *p* < 0.05 2. *p* < 0.05
Ou, 2005	NCNI criteria	Single center RCT	QJJLY+PB	Placebo+PB	36	6/12; 32.06 ± 12.40	7/11; 30.70 ± 10.51	N.R.	N.R.	2m	1. Clinical absolute and relative scores 2. Clinical efficacy	1. *p* < 0.05 2. *p* < 0.05
Ju, 2003	Home made criteria	Single center RCT	TW+ PDN	PDN	40	6/14; 27.80 ± 5.40	8/12; 29.70 ± 8.51	N.R.	N.R.	4m	1. Clinical absolute and relative scores 2. Clinical efficacy 3. Relapse 4. Side effect	1. *p* < 0.05 2. *p* < 0.05 3. *p* < 0.05
Shuang and Tan, 2014	CMAN criteria	Single center RCT	YQCS+ PDN and PB	placebo+ PDN and PB	38	8/12; 22–60	7/11; 20–59	N.R.	N.R.	2.5m	1. Clinical efficacy 2. Side effect	1. *p* > 0.05
Zu, 2015	Home made standard	Single center RCT	BZYQ+ PDN	PDN	50	N.R.	N.R.	N.R.	N.R.	2m	1. Clinical efficacy 2. Side effect	1. *p* > 0.05 2. *p* < 0.05
Lai, 2013	NCNI criteria	Single center RCT	BPQL+ PDN	PDN	60	14/16; 7–71	15/15; 8–74	3.00 ± 2.80 (y)	3.80 ± 3.70 (y)	3m	1. Clinical absolute scores 2. Clinical efficacy 3. Relapse 4. Side effect	1. *p* < 0.05 2. *p* > 0.05 3. *p* < 0.05
Liang, 2011	NCNI criteria	Single center RCT	JLK+ PB	PB	30	8/7; 19–64	5/10; 20–60	N.R.	N.R.	6m	1. Clinical absolute and relative scores 2. Clinical efficacy 3. Relapse	1. *p* < 0.05 2. *p* < 0.05 3. *p* < 0.05
Li, 2012	NCNI criteria	Single center RCT	YQQSF	PB	40	12/8; 38.50 ± 11.30	10/10; 40.25 ± 11.31	N.R.	N.R.	2m	1. QMG scores 2. Clinical efficacy 3. Side effect	1. *p* < 0.01 2. *p* < 0.05 3. *p* > 0.05
Wang et al., 2008	Home made standard	Single center RCT	FYYJ+ PB	Placebo+ PB	120	35/25; 40.10 ± 7.30	34/26; 39.80 ± 5.40	N.R.	N.R.	3m	1. Clinical efficacy 2. Relapse	1. *p* < 0.01 2. *p* < 0.01
Xu, 2006	NCNI criteria	Single center RCT	ZJL+ placebo	placebo+ PDN	60	12/18; 18–64	11/19; 19–63	N.R.	N.R.	3m	1. Clinical absolute and relative scores 2. Clinical efficacy 3. Side effect	1. *p* > 0.05 2. *p* > 0.05
Xu et al., 2004	NCNI criteria	Single center RCT	ZJL+ placebo	placebo+ PDN	144	37/35 38.82 ± 17.23	34/38 39.11 ± 18.19	N.R.	N.R.	3m	1. Clinical efficacy 2. Relapse 3. Side effect	1. *p* > 0.05 2. *p* < 0.05
Bao et al., 2016	NCNI criteria	Multi-center RCT	HQFF+ PB	Placebo+ PB	241	60/61 44.51 ± 17.25	50/70 48.14 ± 17.04	26.17 ± 12.35 (m)	23.53 ± 11.90 (m)	3m	1. Clinical absolute scores 2. Clinical efficacy	1. *p* < 0.01 2. *p* < 0.01
Bao, 2016	NCNI criteria	Single center RCT	HQFF+ PB	Placebo+ PB	60	13/17 44.5 ± 16.83	15/15 47 ± 17.02	26.17 ± 12.35 (m)	23.53 ± 11.90 (m)	9m	1. Clinical absolute scores 2. Clinical efficacy 3. Side effect	1. *p* < 0.05 2. *p* < 0.05

*NCNI criteria, 5th National Conference of Neuroimmunology Criteria; CMAN criteria, Chinese Medical Association of Neurology criteria; PDN, Prednisone; PB, pyridostigmine bromide; y, year; N.R., not reported; HQFF, Huangqi Fufang granule; JJN, JianJiNing granule; QJJLY, Qiangji Jianli Yin; TW, Tan Wei Capsule; YQCS, Yiqi Chushi Recipe; BZYQ, Buzhong Yiqi decoction; BPQL, Bupi Qiangli Compound; JLK, Ji Li Kang Drinking; YQQSF, Yiqi Qushi Fang; FYYJ, Fuyuan Yiji Capsule; ZJL, Zhongjiling Tablet; RCT, randomized-controlled clinical trials; m, month.*

**Table 2:** Ingredients and usage of CHM.

Included studies	Prescription name	Ingredients of herb prescription	Usage of prescription	Preparations
Niu, 2009	HQFF	Radix Astragali seu Hedysari 50 g, Radix Pseudostellariae 25 g, Rhizoma Atractylodis Macrocephalae 15 g, Rhizoma Cimicifugae 10 g, Radix Saposhnikoviae 10 g, Radix Angelicae Sinensis 10 g, Fructus Lycii 15 g, Fructus Corni 15 g	300 g tid po	Granule
Jiang et al., 2014	JJN	Radix Astragali seu Hedysari, Radix Pseudostellariae, Rhizoma Atractylodis Macrocephalae, Fructus Aurantii, Rhizoma Cimicifugae, Herba Leonuri, Radix Saposhnikoviae, Radix Angelicae Sinensis, Fructus Lycii, Radix Polygoni Multiflori, Fructus Corni	210 g bid po	Granule
Ou, 2005	QJJLY	Radix Astragali seu Hedysari, Radix Codonopsis, Rhizoma Atractylodis Macrocephalae, Radix Angelicae Sinensis, Rhizoma Cimicifugae, Radix Bupleuri, Pericarpium Citri, Radix Glycyrrhizae, Cayratia japonica	20 ml tid po	Oral liquid
Ju, 2003	TW	Radix Astragali seu Hedysari, Placenta Hominis, Semen Strychni, Radix Glycyrrhizae	2# tid po	Capsule
Shuang and Tan, 2014	YQCS	Radix Astragali seu Hedysari 30 g, Rhizoma Atractylodis15 g, Rhizoma Atractylodis 15 g, Semen Coicis 30 g, Rhizoma Atractylodis 15 g, Radix Achyranthis Bidentatae 10 g, Semen Arecae 10 g, Fructus Chaenomelis 15 g, Radix Angelicae 15 g, Radix Salviae Miltiorrhizae 15 g, Poria 15 g, Radix Bupleuri 10 g, Radix Glycyrrhizae 10 g	205 g qd po	Granule
Zu, 2015	BZYQ	Pericarpium Citri 15 g, Radix Angelicae Sinensis 10 g, Radix Codonopsis 30 g, Radix Glycyrrhizae 5 g, Rhizoma Cimicifugae 10 g, Rhizoma Atractylodis 15 g, Radix Astragali seu Hedysari 60 g, Radix Bupleuri 10 g	250 ml bid po	Decoction
Lai, 2013	BPQL	Radix Astragali seu Hedysari 60 g, Radix Codonopsis 20 g, Rhizoma Atractylodis Macrocephalae 15 g, Radix Angelicae Sinensis 12 g, Herba Epimedii 15 g, Radix Aconiti Lateralis Preparata 40 g, Rhizoma Smilacis Glabrae 20 g	200/3 ml tid po	Decoction
Liang, 2011	JLK	Radix Astragali seu Hedysari, Radix Codonopsis, Rhizoma Atractylodis Macrocephalae, Semen Coicis, Radix Angelicae Sinensis, Rhizoma Cimicifugae, Radix Bupleuri, Cayratia japonica, Radix Polygoni Multiflori Preparata	200 ml bid po	Oral liquid
Li, 2012	YQQSF	Radix Astragali seu Hedysari 60 g, Radix Ginseng 15 g, Rhizoma Atractylodis Macrocephalae 15 g, Radix Angelicae Sinensis 15 g, Rhizoma Atractylodis 12 g, Rhizoma Alismatis 12 g, Rhizoma Cimicifugae 9 g, Cortex Phellodendri 9 g	200 ml bid po	Decoction
Wang et al., 2008	FYYJ	Radix Ginseng, Cornu Cervi Pantotrichum, Rhizoma Atractylodis Macrocephalae, Poria, Radix Rehmanniae Preparata, Radix Bupleuri, Rhizoma Ligustici Chuanxiong, Rhizoma Acori Tatarinowii, Radix Gentianae, Radix Glycyrrhizae	8# tid po	Capsule
Xu, 2006	ZJL	Cornu Cervi Pantotrichum, Radix Ginseng, Semen Cuscutae, Radix Astragali seu Hedysari, Fructus Lycii, Radix Angelicae Sinensis, Herba Ephedrae, Herba Epimedii, Placenta Hominis, Rhizoma Atractylodis, Poria	4# tid po	Tablet
Xu et al., 2004	ZJL	Cornu Cervi Pantotrichum, Radix Ginseng, Semen Cuscutae, Radix Astragali seu Hedysari, Fructus Lycii, Radix Angelicae Sinensis, Herba Ephedrae, Herba Epimedii, Placenta Hominis, Rhizoma Atractylodis, Poria	4# tid po	Tablet
Bao et al., 2016	HQFF	Radix Astragali seu Hedysari, Rhizoma Cimicifugae, Radix Saposhnikoviae, Rhizoma Atractylodis Macrocephalae, Radix Bupleuri, Radix Angelicae Sinensis, Fructus Lycii	10 g tid po	Granule
Bao, 2016	HQFF	Radix Astragali seu Hedysari, Rhizoma Cimicifugae, Radix Saposhnikoviae, Rhizoma Atractylodis Macrocephalae, Radix Bupleuri, Radix Angelicae Sinensis, Fructus Lycii	10 g tid po	Granule


*HQFF, Huangqi Fufang granule; JJN, JianJiNing granule; QJJLY, Qiangji Jianli Yin; TW, Tan Wei Capsule; YQCS, Yiqi Chushi Recipe; BZYQ, Buzhong Yiqi decoction; BPQL, Bupi Qiangli Compound; JLK, Ji Li Kang Drinking; YQQSF, Yiqi Qushi Fang; FYYJ, Fuyuan Yiji Capsule; ZJL, Zhongjiling Tablet. bid, bis in die; d, day; po, peros; qd, quaquedie; tid, ter in die; #, tablet.*

### Risk of Bias and Quality of Studies

The RoB was assessed by using the Cochrane RoB tool. Among the 14 included studies, the number of criteria varied from 4/7 to 7/7. All of the included studies were reported random allocation, majority studies (12/14) had described the concrete method of random sequences generation, while the remaining 2 studies had no details. Two studies mentioned the concealment allocation. Eight studies reported the blinding, including three double-blind and five single-blind; the remaining studies neither mentioned the blinding nor provided any other information to assess the blinding (unclear). All studies met the criterion of incomplete outcome data as drop-out data or no drop-out patients were reported specifically. Moreover, baseline comparisons were well performed, while none had sample size estimate. More details about RoB assessment of each trial were presented in **Table 3**.

**Table 3:** The Cochrane Collaboration’s tool for assessing RoB.

	A	B	C	D	E	F	G	H
Niu, 2009	+	+	+	+	+	+	+	7+
Jiang et al., 2014	+	+	?	?	+	−	+	4+
Ou, 2005	+	?	+	+	+	?	+	5+
Ju, 2003	+	?	−	−	+	+	+	4+
Shuang and Tan, 2014	+	?	+	+	+	+	+	6+
Zu, 2015	+	?	−	−	+	+	+	4+
Lai, 2013	+	?	−	−	+	+	+	4+
Liang, 2011	+	?	−	−	+	+	+	4+
Li, 2012	+	?	−	−	+	+	+	4+
Wang et al., 2008	?	?	+	−	+	+	+	4+
Xu, 2006	+	−	+	+	+	+	+	6+
Xu et al., 2004	?	?	+	?	+	+	+	4+
Bao et al., 2016	+	?	+	−	+	?	+	4+
Bao, 2016	+	?	+	−	+	+	+	5+

*A, adequate sequence generation; B, concealment of allocation; C, blinding of participants and personnel; D, blinding of outcome assessment; E, incomplete out-come data; F, selective reporting; G, other bias.*

### Effectiveness

#### CHM vs. Placebo

None of RCTs used a specific comparison between CHM and placebo.

#### CHM vs. WCM

**FIGURE 2:**
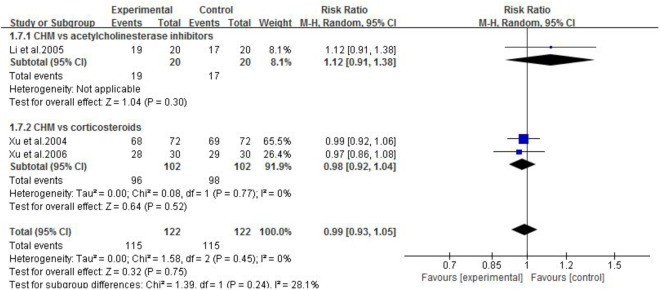
The forest plot: The total clinical effective rate of CHM vs. WCM.

**FIGURE 3:**
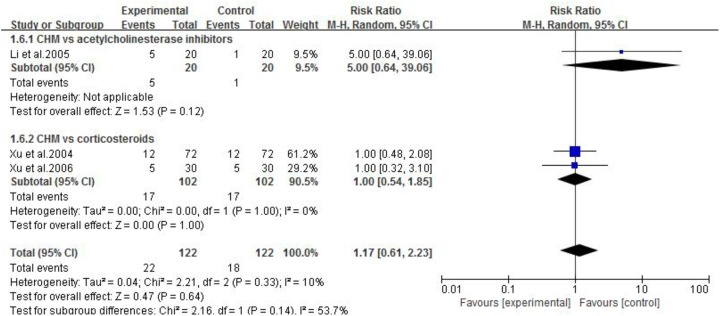
The forest plot: Recovery rate of CHM vs. WCM.

Three studies (Xu et al., 2004; Xu, 2006; Li, 2012) compared CHM with WCM. One study (Li, 2012) showed that CHM was superior to pyridostigmine bromide according to QMG scores. The other two studies (Xu et al., 2004; Xu, 2006) showed no significant difference between CHM and corticosteroids according to MG clinical absolute and relative scores. Meta-analysis of three studies (Xu et al., 2004; Xu, 2006; Li, 2012) showed no significant difference in total clinical effective rate (*n* = 244, RR 0.99, 95% CI: 0.93–1.05, *p* = 0.75; heterogeneity χ^2^ = 1.58, *df* = 2, *p* = 0.45, *I*^2^ = 0%, **Figure 2**) and recovery rate (*n* = 244, RR 1.17, 95% CI: 0.61–2.23, *p* = 0.64; heterogeneity χ^2^ = 2.21, *df* = 2, *p* = 0.33, *I*^2^ = 10%, **Figure 3**) comparing CHM with pyridostigmine bromide or corticosteroids.

#### CHM Plus WCM vs. WCM

**FIGURE 4:**
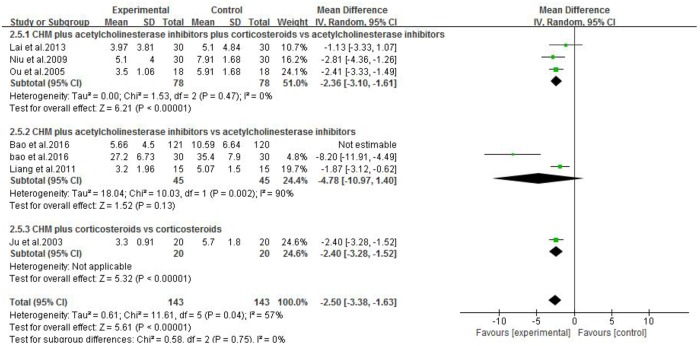
The forest plot: MG clinical absolute and relative scores of CHM plus WCM vs. WCM.

**FIGURE 5:**
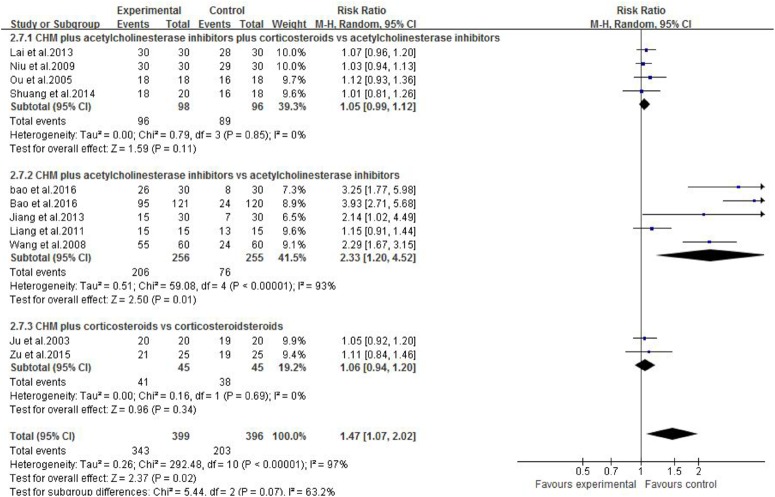
The forest plot: The total clinical effective rate of CHM plus WCM vs. WCM.

**FIGURE 6:**
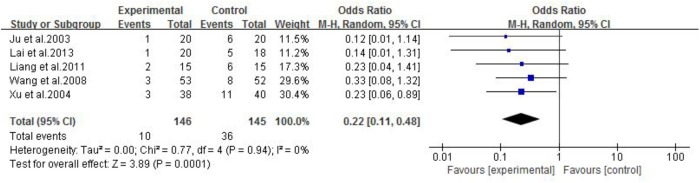
The forest plot: Relapse rate after follow-up of CHM plus WCM vs. WCM.

Eleven studies were included. One study (Jiang et al., 2014) showed that Jianjining Granule paratherapy significantly improved QMG score at both 3 and 6 months compared to pyridostigmine bromide (*p* < 0.05). Meta-analysis of three studies (Ou, 2005; Niu, 2009; Lai, 2013) showed that CHMs paratherapy were significant for decreasing MG clinical absolute and relative score (*n* = 156, MD −2.36, 95% CI: −3.10 to −1.61, *p* < 0.00001; heterogeneity χ^2^ = 1.53, *df* = 2, *p* = 0.47, *I*^2^ = 0%, **Figure 4**) compared to pyridostigmine bromide and corticosteroids; one (Ju, 2003) study also have positive result compared with corticosteroids (*p* < 0.01); three studies (Barohn et al., 1998; Bao, 2016; Bao et al., 2016) failed to pool analysis because of high heterogeneity and all showed statistical significant difference compared with pyridostigmine bromide (*p* < 0.01). Meta-analysis of four studies (Ou, 2005; Niu, 2009; Lai, 2013; Shuang and Tan, 2014) showed CHMs paratherapy significantly improved the total clinical effective rate (*n* = 194, RR 1.05, 95% CI: 0.99–1.12, *p* = 0.11; heterogeneity χ^2^ = 0.79, *df* = 3, *p* = 0.85, *I*^2^ = 0%, **Figure 5**) compared with pyridostigmine bromide and corticosteroids; two studies (Ju, 2003; Zu, 2015) are similar result (*n* = 90, RR 1.06, 95% CI: 0.94–1.20, *p* = 0.34; heterogeneity χ^2^ = 0.16, *df* = 1, *p* = 0.69, *I*^2^ = 0%) compared with corticosteroids; five studies (Wang et al., 2008; Liang, 2011; Jiang et al., 2014; Bao, 2016; Bao et al., 2016) failed to pool analysis because of high heterogeneity and all showed statistical significant difference compared with pyridostigmine bromide (*p* < 0.01). Meta-analysis of five studies (Ju, 2003; Xu et al., 2004; Wang et al., 2008; Liang, 2011; Lai, 2013) showed that CHMs paratherapy were significant for improving the relapse rate after follow-up (*n* = 291, OR 0.22, 95% CI: 0.11–0.48, *p* = 0.0001; heterogeneity χ^2^ = 0.77, *df* = 4, *p* = 0.94, *I*^2^ = 0%, **Figure 6**) compared WCM controls.

### Adverse Event

Adverse effects were reported in 10 studies, and the remaining four (Ou, 2005; Wang et al., 2008; Liang, 2011; Bao et al., 2016) studies did not mention. Two (Niu, 2009; Jiang et al., 2014) studies reported that no adverse events occurred during treatment. Eight (Ju, 2003; Xu et al., 2004; Xu, 2006; Li, 2012; Lai, 2013; Shuang and Tan, 2014; Zu, 2015; Bao, 2016) studies reported detailed information of adverse effects with 4/247 patients in trial group vs. 51/245 patients in control group. The frequently occurring adverse events are liver function abnormalities, high fasting glucose, hormonal obesity, infection, gastrointestinal discomfort, granulocytopenia, insomnia, skin allergy, thrombocytopenia, and femoral head necrosis. In particular, 0/247 patients suffered hormonal obesity in the trial groups and 21/245 patients did so in control groups. The majority of adverse effects were mild, and serious adverse events such as life-threatening were not found in included studies.

### Description of the CHMs

A total of 11 herbal decoctions were used in 14 included studies. The number of herbal components in the formulae varied from 4 to 13. The most frequently used herbs across all formulae were listed as follows: milkvetch root (Radix Astragaliseu Hedysari/Astragalus membranaceus), largehead atractylodes rhizome (Rhizoma Atractylodis Macrocephalae), Chinese angelica (Radix Angelicae Sinensis), largetrifoliolious bugbane rhizome (Rhizoma Cimicifugae), Chinese thorowax root (Radix Bupleuri), liquorice root (Radix Glycyrrhizae), tangshen (Radix Codonopsis), barbary wolfberry fruit (Fructus Lycii), Indian bread (Poria), divaricate saposhnikovia root (Radix Saposhnikoviae), ginseng (Radix Ginseng) (**Table 4**).

**Table 4:** Frequently used herbs in included studies.

Chinese name	English name	Latin name	Family	Number of studies (%)
Hangqi	Milkvetch root	Radix Astragali seu Hedysari/Astragalus membranaceus	Astragalus Linn.	13 (92.9)
Baizhu	Largehead atractylodes rhizome	Rhizoma Atractylodis Macrocephalae	Atractylodes	13 (92.9)
Danggui	Chinese angelica	Radix Angelicae Sinensis	Angelica L.	12 (85.7)
Shengma	Largetrifoliolious bugbane rhizome	Rhizoma Cimicifugae	Cimicifuga	8 (57.1)
Chaihu	Chinese thorowax root	Radix Bupleuri	Bupleurum L.	7 (50.0)
Gouqizi	Barbary wolfberry fruit	Fructus Lycii	Lycium	6 (42.9)
Gancao	Liquorice root	Radix Glycyrrhizae	Glycyrrhiza Linn.	5 (35.7)
Dangshen	Tangshen	Radix Codonopsis	Codonopsis Wall.	4 (28.6)
Fangfeng	Divaricate saposhnikovia root	Radix Saposhnikoviae	Saposhnikovia Schischk	4 (28.6)
Fuling	Indian bread	Poria	Wolfiporia Ryv.&Gilbn	4 (28.6)
Renshen	Ginseng	Radix Ginseng	Panax L.	4 (28.6)

### Possible Mechanisms

The pharmacological effects of active compounds of frequently used herbs on MG and related autoimmune disease are as follows: (1) Astragalus membranaceus: promoting the expression of transcription factor Forkhead box protein P3 (FoxP3) to up-regulate T regulatory cells (Tregs) (Qu et al., 2010; Jin et al., 2013), and decreasing cytokine expression such as IL-4 and IL-13 (Chen et al., 2014b; Zhao et al., 2016); (2) Radix Ginseng: increasing number of Tregs and inhibiting Th17 cell differentiation (Bae et al., 2012; Chen et al., 2014a; Jhun et al., 2014; Lee et al., 2015; Chen et al., 2016); (3) Bupleurum polysaccharides (BPs) from Radix Bupleuri: a decrease of autoantibodies and immunoglobulin G (IgG) (Wang et al., 2009; **Table 5**). (4) Huperzine A (HupA), isolated from Huperzia serrata and flavonoid derivatives from Buzhongyiqi Decoction exhibited anti-acetylcholinesterase effects (Orhan, 2013; Cui et al., 2015).

**Table 5:** Characteristics of mechanism studies.

Included studies	Active ingredients	Herb source	Strain, model (n/n)	Experimental group	Control group	Possible mechanisms (signaling pathway)
Qu et al., 2010	Astragaloside	Astragalus membranaceus	BALB/c mice, Allografted model	AMI (60 g/kg, i.g. ) for 14 d	NS (volume-matched, i.g.) for 14 d	Up-regulate the Treg ratio and promote Foxp3 expression
Jin et al., 2013	/	Astragalus membranaceus	SD rats, asthma model (10/10/10/10)	AM (2.5, 5.0, or 10.0 g/kg, p.o.) for 42 d	PBS (volume-matched, p.o.) for 42 d	Increase population of CD4+CD25+Foxp3+ Treg cells and promote Foxp3+ mRNA expression
Chen et al., 2014b	/	Astragalus membranaceus	C57BL/6JNarl mice, asthma model	AM (3 μg/kg, p.o.) for 8 d	NS (volume-matched, p.o.) for 8 d	Increase the activity of PPARγ to decrease the expression of Th2 cytokines
Zhao et al., 2016	Astragalus polysaccharide	Astragalus membranaceus	SD rats, colitis model (8/8)	APS (400 mg/kg, p.o.) for 7 d	NS (volume-matched, p.o.) for 7 d	Improve the level of Treg cells, regulate cytokine expression
Bae et al., 2012	Ginsenoside Rp1	Radix Ginseng	C57BL/6 mice, LPS-induced inflammation model (6/7)	G-Rp 1 (10 mg/kg, p.o.) for 7 d	PBS (volume-matched, i.g.) for 7 d	Increase in Tregs is due to the increase of Treg survival and the conversion of memory type Tregs rather than the generation of new Tregs.
Chen et al., 2014a	Ginsenoside metabolite compound K	Radix Ginseng	SD rats, rheumatoid arthritis model	CK (10, 40, and 160 mg/kg, p.o.) for 33 d	NS (volume-matched, p.o.) for 33 d	Decrease of activated T cells and the increase of naïve T cells and Treg
Jhun et al., 2014	Ginsenoside Rb1	Radix Ginseng	DBA/1J mice, rheumatoid arthritis model	RGE (10 mg/kg, p.o.) for 42 d	NS (volume-matched, p.o.) for 42 d	Increase the number of Treg cells and decrease the number of T17 cells
Lee et al., 2015	Ginsenoside Rg1 Ginsenoside Rh1, 20(S)-protopanaxatriol	Radix Ginseng	ICR mice, colitis model	Ginsenoside Rg1 (20 mg/kg, p.o.) Ginsenoside Rh1 (20 mg/kg, p.o.) 20(S)-protopanaxatriol (10 and 20 mg/kg, p.o.) for 3 d	Vehicle (volume-matched, p.o.) for 3 d	Inhibit Th17 cell differentiation, induced to Treg cell differentiation
Chen et al., 2016	Ginsenoside Rb1	Radix Ginseng	Lewis rats, MG model (7/7/7)	G-Rb 1 (2.6 and 3.7 mg/kg, p.o.) for 37 d	Vehicle (volume-matched, p.o.) for 37 d	Increase the percentage of Treg cells, up-regulated the expression of IL-10 and IL-4
Wang et al., 2009	Bupleurum polysaccharides	Radix Bupleuri	BALB/c mice, Lupus nephritis model	BPs (30 and 15 mg/kg, p.o.) for 35 d	Vehicle (volume-matched, p.o.) for 35 d	Decrease autoantibodies and immunoglobulin G (IgG)

*AMI, Astragalus Membranaceus Injection; PBS, phosphate-buffered saline; LPS, lipopolysaccharide; RGE, red ginseng extracts; G-Rb1, Ginsenoside Rb1; DM, diabetes mellitus; NS, normal saline; APS, Astragalus polysaccharide; G-Rp1, Ginsenoside Rp1; CK, Ginsenoside metabolite compound K; BPs, Bupleurum polysaccharides.*

## Discussion

### Summary of Evidence

Fourteen high-quality RCTs with 1039 individuals were identified for analysis. The findings demonstrated that CHM as adjuvant therapy for MG could reduce the QMG scores or MG clinical absolute and relative scores, reduce relapse rate, and improve total clinical effective rate. There were fewer adverse effects in comparison with controls. The possible mechanisms are associated with immunoregulation by reconstituting of the functional ability of Tregs. Thus, the present evidence supports, at least to an extent, that CHM can be recommended for routine use for MG patients.

### Limitations

Although the high-quality RCTs were included, there were still some methodological weaknesses in the primary studies. First, only two studies reported the allocation concealment. A trial with inadequate or unclear concealment of allocation is likely to overestimate the therapeutic effect (Higgins and Green, 2011). Second, blinding was available to reduce the occurrence of performance bias and ascertainment bias in clinical trials (Lewis, 2003). Eight studies used the blinding, whereas only four studies used double-dummy, placebo design. Placebo plays a crucial role as a control in RCTs. A placebo effect is conceptually defined as the beneficial effect associated with an intervention that does not include the presumed active ingredients. Thus, placebo-controlled randomized trials are well-recognized method when evaluating the efficacy of clinical treatment. In the present study, none of RCTs used a direct comparison between CHM and placebo, while several RCTs adopted double-dummy, placebo-controlled trial. One of the main reasons is permissible to use placebo and ethically acceptable only on specific occasions (Millum and Grady, 2013). In addition, the placebo of CHMs was difficult to prepare in the same color, flavor, and taste. Third, most of trials are without calculating the formal pretrial sample size. The trials with inadequate sample sizes seem to be one risk in exaggerating intervention benefits. Finally, owing to highly variable in composition and dosage of CHMs, it is difficult to assess the efficacy of a specific CHM by performing a pooling analysis (Lewis, 1999).

### Implications for Practice

The use of CHMs in the treatment of MG has increased in the past decades. However, the choice of CHMs is empirical and lacking consensus among clinic doctors. In this study, the findings demonstrated that CHM as adjuvant therapy with WCM could improve MG symptoms. The most frequently used herbs such as Astragalus membranaceus and Radix Ginseng should be considered further in the development of Chinese herbal prescription for MG. Thus, following on the treatment principle according to the high-frequency use of CHMs for MG, their treatment principles can guide to CHM treatment for MG and thus enhance the clinical effectiveness and safety.

### Implications for Research

Some methodological weaknesses also existed in the primary trials. Thus, we recommend that CONSORT 2010 statement (Schulz et al., 2010), CONSORT for CHM Formulas (Cheng et al., 2017), and RCTs investigating CHM (Flower et al., 2012) should be used as the guidelines when the designing, registering, and reporting of further RCTs.

Naive CD4^+^ T cells can differentiate into both anti-inflammatory Tregs and proinflammatory IL-17-producing T (Th17) cells, Th1, and Th2 (Masuda et al., 2010). Tregs play a key role in immunologic tolerance. The transcription factor FoxP3 is selectively expressed in Tregs, which is the key modulators of Tregs activation and function (Marson et al., 2007). Tregs actively mediate self-tolerance and thus control autoimmunity by suppressing the activation, proliferation, and effector function such as a cytokine production of various immune cells (Sakaguchi et al., 2008; Alahgholi-Hajibehzad et al., 2015b). Conversely, Th17 cells play a crucial role in the initiation and maintenance of autoimmune tissue injury (Schaffert et al., 2015). The disequilibrium of Th17 and Tregs is involved in the pathogenesis of various autoimmune diseases (Villegas et al., 2018). The numerical, functional, and migratory deficits of Tregs through cognate interactions with B cells lead to the synthesis of anti-AChR antibodies associated with MG pathogenesis (Danikowski et al., 2017). Thus, reconstitution of the Tregs disfunction or inhibition of the Th17 cells may be potential targets for MG treatment (Alahgholi-Hajibehzad et al., 2015a; Aricha et al., 2016). Cytokine networks affected the balance of Th1, Th2, Tregs, and Th17 cell subsets. IL-10 and IL-4 produced by Th2 cells, serve as a protective role in MG. It was suggested that IL-10 and IL-4 were acting on the antigen-presenting cell to inhibit cytokine production by Th1 cells and also can inhibit antigen presentation of macrophages (Chen et al., 2016). In addition, IL-4 and IL-10 production increased in the presence of Treg in MG patients (Alahgholi-Hajibehzad et al., 2017).

Mechanisms of CHMs and their active components for MG are gaining attention. Notably, the included trials presented evidence of immunologic regulation effects in multiple models of autoimmune disease as follows: Astragalus membranaceus (Qu et al., 2010; Jin et al., 2013; Zhao et al., 2016), Ginsenoside Rp1, Ginsenoside Rb1, and Ginsenoside metabolite compound K (Bae et al., 2012; Chen et al., 2014a, 2016; Jhun et al., 2014) from Radix Ginseng increase number of Tregs through promoting Foxp3 expression and further an increase of endogenous Treg population, or the adaptive transfer of compatible exogenous and possibly autologous functional Tregs (Bae et al., 2012). Astragalus membranaceus can regulate a cytokine production of various immune cells (Chen et al., 2014b; Zhao et al., 2016). Ginsenoside Rb1 (Jhun et al., 2014), Ginsenoside Rg1, Ginsenoside Rh1, and 20(*S*)-protopanaxatriol (Lee et al., 2015) from Radix Ginseng can inhibit Th17 cell differentiation, and Ginsenoside Rb1 (Chen et al., 2016) regulates the expression of IL-10 and IL-4, BP from Radix Bupleuri reduced autoantibodies and IgG (Wang et al., 2009). HupA is an AChE inhibitor (Orhan, 2013). However, the specific mechanism of CHMs and their active compounds still needs clarity.

## Conclusion

The evidence available from the present study is supported, at least to an extent, that CHMs paratherapy can be recommended for routine use for MG. Furthermore, high frequent uses of CHMs are selected to contribute to composing a herbal formula as a promising candidate for further clinical application and MG trials.

## Author Contributions

G-QZ contributed as the senior author and the principal investigator (PI) of this study, and refined the study. SC, M-BX, and X-LZ wrote the first draft of the manuscript and contributed to the overall design. T-YJ and P-QR identified and reviewed the studies for eligibility, and performed the meta-analysis of the data. All authors read, critically reviewed, and approved the final manuscript.

## Conflict of Interest Statement

The authors declare that the research was conducted in the absence of any commercial or financial relationships that could be construed as a potential conflict of interest.
